# Overexpression of long noncoding RNA *4933425B07Rik* leads to renal hypoplasia by inactivating Wnt/β-catenin signaling pathway

**DOI:** 10.3389/fcell.2023.1267440

**Published:** 2023-10-17

**Authors:** Shanshan Xue, Xuanjin Du, Minghui Yu, Haixin Ju, Lihong Tan, Yaxin Li, Jialu Liu, Chunyan Wang, Xiaohui Wu, Hong Xu, Qian Shen

**Affiliations:** ^1^ Department of Nephrology, Children’s Hospital of Fudan University, Shanghai Kidney Development and Pediatric Kidney Disease Research Center, Shanghai, China; ^2^ Department of Nephrology, Children’s Hospital of Chongqing Medical University, National Clinical Research Center for Child Health and Disorders, Ministry of Education Key Laboratory of Child Development and Disorders, Chongqing, China; ^3^ State Key Laboratory of Genetic Engineering and National Center for International Research of Development and Disease, Institute of Developmental Biology and Molecular Medicine, Collaborative Innovation Center of Genetics and Development, School of Life Sciences, Fudan University, Shanghai, China

**Keywords:** PB transposon, lncRNAs, 4933425B07Rik, CAKUT, renal development, Wnt/β-catenin signaling pathway

## Abstract

Congenital anomalies of the kidney and urinary tract (CAKUT) is a general term for a class of diseases that are mostly caused by intrauterine genetic development limitation. Without timely intervention, certain children with CAKUT may experience progressive decompensation and a rapid decline in renal function, which will ultimately result in end-stage renal disease. At present, a comprehensive understanding of the pathogenic signaling events of CAKUT is lacking. The role of long noncoding RNAs (lncRNAs) in renal development and disease have recently received much interest. In previous research, we discovered that mice overexpressing the lncRNA *4933425B07Rik* (*Rik*) showed a range of CAKUT phenotypes, primarily renal hypoplasia. The current study investigated the molecular basis of renal hypoplasia caused by *Rik* overexpression. We first used Rapid Amplification of cDNA ends (RACE) to obtain the full-length sequence of *Rik* in *Rik* ^*+/+*^;*Hoxb7* mice. Mouse proximal renal tubule epithelial cells (MPTCs) line with *Rik* overexpression was constructed using lentiviral methods, and mouse metanephric mesenchyme cell line (MK3) with *Rik* knockout was then constructed by the CRISPR‒Cas9 method. We performed RNA-seq on the *Rik*-overexpressing cell line to explore possible differentially expressed molecules and pathways. mRNA expression was confirmed by qRT‒PCR. Reduced levels of *Wnt10b*, *Fzd8*, and *β-catenin* were observed when *Rik* was expressed robustly. On the other hand, these genes were more highly expressed when *Rik* was knocked out. These results imply that overabundance of *Rik* might inhibit the Wnt/β-catenin signaling pathway, which may result in renal hypoplasia. In general, such research might help shed light on CAKUT causes and processes and offer guidance for creating new prophylactic and therapeutic strategies.

## 1 Introduction

A wide range of anatomical urinary abnormalities are termed congenital anomalies of the kidney and urinary tract (CAKUT), the majority of which are the result of mistakes in renal growth. The most common defects are vesicoureteral reflux, hydronephrosis, kidney hypoplasia, kidney agenesis, kidney dysplasia, supernumerary kidneys, horseshoe kidney, ureterovesical junction blockage, megaureter, and ureteropelvic junction obstruction (Nicolaou et al., 2015). CAKUT is estimated to affect six out of every 1,000 births worldwide (Murugapoopathy and Gupta, 2020). According to data, CAKUT is one of the most common etiology of chronic renal disease (Harada R et al., 2022). CAKUT therefore imposes a severe economic burden and severe psychological stress in patients and their families.

Mouse renal development is similar to that of humans and mainly involves the pronephros, mesonephros and metanephros, three nephric phases. At embryonic day (E) 10.5 in mice and 4.5 weeks of gestation in humans, the mesonephros are formed when the ureteric bud (UB) starts to develop outward from the Wolffian duct after receiving an induction signal from the metanephric mesenchyme (MM). The original T-shaped building is created at E11.5 (equal to 5 weeks of human gestation) after the MM obtains the UB’s induction signal, starts to aggregate around its tip, and branches to both sides. The cap mesenchyme, or a cluster of MM cells, forms around the branching points of UB and gives rise to nephron progenitors. This entire procedure, known as UB induction, is crucial for kidney development. UB then undergoes repeated proliferation and branching to create new UB tips. At E15.5 (equal to 8 weeks of human gestation), the process starts to slow and ultimately forms an elaborate collecting duct system (Marrone and HO, 2014). Renal hypoplasia results from issues with these complex and precise interactions that are necessary for the production of correct branching morphology between the UB and MM. Renal hypoplasia manifests as a small kidney with fewer nephrons due to a lack of branching morphogenesis. Unilateral hypoplasia occurs per 1,000 births, while bilateral hypoplasia is rarer (1 in 4,000) (Jain and Chen, 2018).

At present, the etiology of CAKUT is not fully understood, and CAKUT pathogenesis may be multifactorial in some cases (Nicolaou et al., 2015). To date, more than 50 CAKUT pathogenic genes have been described in the scientific research field, and each gene explains only approximately 1% of cases in the CAKUT cohort (Hwang et al., 2014; Heidet et al., 2017). All currently known pathogenic genes of CAKUT can explain only approximately 18%–20% of urinary developmental malformations (Madariaga et al., 2013; Sanna-Cherchi et al., 2018). These findings suggest that we need to identify new pathogenic factors, such as epigenetic changes in noncoding RNAs (Huang et al., 2020). Long noncoding RNAs (lncRNAs) are the longest non-protein-coding RNAs. LncRNAs constitute on average only one-tenth of mRNAs and can be divided into different lncRNA classes according to their upstream and downstream positions with respect to nearby genes (Dahariya et al., 2019). Moreover, lncRNAs are widely distributed in cells, and their widespread distribution determines the diversity of their functional mechanisms. In the nucleus, for instance, lncRNAs can play roles in gene architecture, transformation of cell chromatin or chromosome states, and maintenance of the correct splicing process (Yao et al., 2019; Gil and Ulitsky, 2020; Nair et al., 2020). LncRNAs in the cytoplasm can change gene expression levels by controlling key elements in the translation process and can also bind to microRNAs (Fatica and Bozzoni, 2014; Chen et al., 2015). According to reports, lncRNAs are crucial for normal kidney growth, and erroneous play can contribute to renal disease. For example, during the culture of kidney nephron progenitor cells, the lncRNA *GM29418* has been found to regulate *Six2*, a key gene for renal development (Nishikawa et al., 2019). Another article reported abnormal expression of the lncRNA HOXB3OS in individuals with polycystic kidney disease that is autosomal dominant, and *in vitro* studies have confirmed that the lncRNA *Hoxb3os* can regulate the mTOR signaling pathway and mitochondrial respiratory function (Aboudehen et al., 2018).

In our previous study, the lncRNA *4933425B07Rik* (*Rik*) was overexpressed in *Rik* ^*PB/PB*^;*Hoxb7* mice, and the neonatal kidneys showed multiple CAKUT phenotypes, including duplicated kidneys, hydronephrosis, kidney hypoplasia, kidney dysplasia, and vesicoureteral reflux, the most common of which was kidney hypoplasia. A reduction in the quantity of UB tips was observed in *Rik* ^*PB/PB*^;*Hoxb7* mouse embryonic kidney cultures *in vitro*; therefore, *Rik* appeared to participate in renal development, and its overexpression led to abnormal renal development (Tan et al., 2021). Thus far, there have been few studies on *Rik* aside from our previous phenotypic study. Therefore, in this study, we constructed cell models of *Rik* overexpression and *Rik* knockout *in vitro* and used *Rik* ^*PB/PB*^;*Hoxb7* mice to examine the underlying molecular mechanisms of kidney hypoplasia induced by *Rik* overexpression.

## 2 Materials and methods

### 2.1 Mice

The piggyBac (PB) transposon, an insertional mutagenesis technique, was used to produce *Rik* ^*PB/+*^ mice on the FVB/NJ genetic background (Tan et al., 2021). The offspring of mated *Rik* ^*PB/+*^ mice and *Hoxb7*/myr-Venus mice were known as *Rik* ^*PB/+*^;*Hoxb7*/myr-Venus (abbreviated *Rik* ^*PB/+*^;*Hoxb7*). For the purpose of producing *Rik* ^*+/+*^;*Hoxb7* mice and *Rik* ^*PB/PB*^;*Hoxb7* mice, *Rik* ^*PB/+*^;*Hoxb7* mice were interbred. The primers utilized for genotyping are listed in Supplementary Table S1. At five o’clock in the afternoon, male and female mice (1:1) were mated in cages; the next morning, when a plug was found, the stage was designated E0.5. All of the experimental mice were specifically pathogen-free (SPF), kept at a temperature of 18°C–22°C, with a humidity level of 50%–60% and light hours of 8 am to 8 pm. The animal study protocols were approved by the Institute of Developmental Biology and Molecular Medicine of Fudan University (Protocol Approval No. SYXK (hu) 2020-0011).

### 2.2 Cell culture

Mouse proximal renal tubule epithelial cells (MPTCs) line was donated by Prof. Aihua Zhang. The mouse metanephric mesenchyme cell line (MK3) was purchased from BLUEFBIO (Shanghai, China). Eagle medium modified with Dulbecco’s and Nutrient Mixture F-12 (Thermo, United States) was used to cultivate MPTCs, while high-glucose DMEM (HyClone, United States) was used to cultivate MK3 cells. They were seeded in a plate that had been precoated with poly-L-lysine (Beyotime Biotechnology, China). Ten percent fetal bovine serum (Thermo, United States) and one unit of penicillin‒streptomycin (Thermo, United States) were added to all media as supplements. An atmosphere of 5% CO_2_ and 37°C was used to incubate the cells.

### 2.3 Rapid amplification of cDNA ends (RACE)

The sample for RACE was tested from eight-week-old *Rik* ^*+/+*^;*Hoxb7* mice. Using a TRIzol^®^ Plus RNA Purification Kit (Thermo, United States), total RNA was collected from testicular tissue as directed by the manufacturer. 5′ RACE and 3′ RACE were performed according to the manufacturer of the GeneRacer™ Kit (Thermo, United States). For PCR, the following gene-specific primers (*Rik*) were used: 5′ RACE *Rik*-R1 (5′-GCT​AGG​GTC​CTG​TGA​AAA​ACC​GAG​GAA-3′), 5′ RACE *Rik*-R2 (5′-GGA​CAT​CCA​ATC​CAG​CCT​CCA​CCT​CAG​T-3′), 3′ RACE *Rik*-F1 (5′-GGC​AGC​ACA​CAC​ATT​TAA​TCT​CAG​CAC​TCA-3′), and 3′ RACE *Rik*-F2 (5′-GTA​GTG​AAC​TTT​CTC​AAA​AAC​CAA​ACC​AAA​C-3′).

### 2.4 Fertility assessment

The farrowing rate and litter size of the mice in each group were quantified to preliminarily assess the reproductive capacity of *Rik* male mice (approximately 7 days after birth). The mice were grouped as follows: *Rik* ^*+/+*^;*Hoxb7* male and female inbred mice composed one group, and *Rik* ^*PB/PB*^;*Hoxb7* male mice mated with *Rik* ^*+/+*^;*Hoxb7* female mice composed the other group. Each group consisted of three cages of mice. The age of the mating male and female mice was 45–60 days. Each cage contained only one male and one female mouse. The male and female mice were caged at 5 o’clock. The mice were observed for 3 months.

### 2.5 Sperm counting and semen morphological analysis

The sperm count was the number of sperm in a semen sample excreted at one time as examined under a microscope. The sperm count value was multiplied by the amount of semen to obtain the amount of sperm ejaculated at one time. *Rik* ^*+/+*^;*Hoxb7* and *Rik* ^*PB/PB*^;*Hoxb7* male mice at 3 months of age were selected. After the mice were rapidly euthanized, testicles were gently removed to a small dish (Thermo, United States) with 1 mL of PBS (Sangon Biotech, China), and the epididymis was repeatedly poked with a 1 mL needle (Kindly Group, China) to help the sperm enter the PBS liquid. Then, the sperm were transferred to a cell microscope (Leica, Germany) for counting. The semen remaining after counting was used for the smear. Thirty microliters of the aforementioned sperm suspension were drawn onto a glass slide (Sangon Biotech, China), and the film was quickly pushed with a coverslip (Sangon Biotech, China) with the slide at an angle of 45° to create a semen smear. Then, the semen smear was placed horizontally for 10 min, dried, immersed in a solution of 95% ethanol and methanol for 2–3 min, removed, and dried again. A microscope image of sperm morphology was captured.

### 2.6 Hematoxylin-eosin (HE) staining

Testicular and epididymal tissues were removed from *Rik* ^*+/+*^;*Hoxb7* and *Rik* ^*PB/PB*^;*Hoxb7* male mice and placed into fixative solution (Servicebio, China). Then, alcohol dehydration, xylene clearing, paraffin immersion, embedding, sectioning, patching, dewaxing and staining, dehydration, clearing, and sealing were performed. The specific experimental procedures have been described previously (Feldman and Wolfe, 2014).

### 2.7 RNA fluorescence *in situ* hybridization (RNA-FISH)

Sangon Biotech in China provided lncRNA-*Rik* probes that were Cy3-labeled. As per the guidelines provided by the manufacturer, hybridizations were performed using a FISH Kit (RiboBio, China). Slices of kidney tissue were briefly fixed with 4% paraformaldehyde (Sangon Biotech, China), followed by 0.5% Triton (Sangon Biotech, China) treatment. The slices were subsequently incubated with a lncRNA-*Rik* probe overnight. All fluorescence pictures were taken with a microscope (Nikon Instruments Inc., Japan). 5′-UGU​GCU​GCC​AUA​UCA​GGC​UCU​AAA​UUC​UAA​CC-3′ was the sequence for lncRNA-*Rik* probes.

### 2.8 Construction of the MPTCs line with *Rik* overexpression by lentivirus

The MPTCs line is most prevalent in the kidney and is a typical cell line used in kidney function and kidney disease studies (Lu et al., 2021). We found that *Rik* had very low background expression in MPTCs, so we deemed MPTCs suitable to construct a *Rik* overexpression cell model. The *Rik* cDNA was amplified and cloned to construct the GV367-Ubi-MCS-SV40-EGFP-IRES-puromycin vector (GeneChem, China), and the correct sequence of *Rik* was verified by sequencing. Briefly, when cell confluence reached 20%–30%, MPTCs were infected in the presence of 1×10^6^ TU/mL lentivirus, and the media were supplemented with 5 mg/mL Polybrene (GeneChem, China). After 16 h, the cells were rinsed with fresh complete medium. The experimental technique properly adhered to all infection control measures. The cells were infected for up to 3 days and then screened with puromycin (Beyotime Biotechnology, China). After screening for at least 1 week, the extent of *Rik* expression was validated using qRT‒PCR. Cryopreservation and subsequent experimental studies were conducted on overexpressed cloned cells.

### 2.9 RNA-seq and data analysis

TRIzol (Thermo Fisher Scientific, United States) was used to collect RNA. A NanoDrop One AZY1705135 (Thermo, United States) was used to detect RNA purity, accuracy, and integrity, and samples with an integrity number greater than 7 were accepted. Magnetic beads (Sangon Biotech, China) were used to screen polyA mRNA, and the mRNA was fragmented with reagent (Sangon Biotech, China). The first and second strands of cDNA were synthesized, and the cDNA was end-repaired and purified. Indexes were affixed for differentiation, and PCR enrichment libraries were constructed. The libraries were subjected to quality inspection and machine sequencing. Then, paired-end reads were generated by sequencing these libraries. Trimmomatic was used for the initial processing of the raw sequencing data (raw reads). The raw reads were processed to generate clean reads in order to improve the reliability of the subsequent analyses. Then, the clean reads were mapped to the reference genome. Depending on the alignment information, the clean reads were assembled into transcripts to measure the expression of genes or transcripts.

### 2.10 Gene ontology (GO) enrichment and kyoto encyclopedia of genes and genomes (KEGG) pathway analysis

GO and KEGG enrichment analyses were used to determine the transcriptional profiles obtained by RNA-seq and to highlight the functional roles of genes with varying expression. Each gene had one or more GO terms. The hypergeometric distribution relationship of these differentially expressed genes with one or more specific branch(es) in the GO classification was calculated. Then, a *p*-value was returned for each differentially expressed gene. A more enriched differentially expressed gene had a lower *p*-value. Hierarchical clustering analysis of genes with differing expression was performed to examine the patterns of transcript expression.

### 2.11 Quantitative real-time polymerase chain reaction (qRT‒PCR)

First, under RNase-free conditions, RNA was extracted from tissue or cell samples. The RNA quality was detected, and the passing RNA was immediately reverse-transcribed into cDNA. Primers were designed to amplify fragments by real-time PCR. The total amount of product after each PCR cycle was determined with fluorescent reagent. Finally, the expression of each particular DNA sequence in the sample was normalized to that of an internal reference. Primers are listed in Supplementary Table S2.

### 2.12 Construction of the MK3 cell line with *Rik* knockout by the CRISPR‒Cas9 method

The MK3 cell line, prior to UB induction, is a representation of the early renal mesenchyme and is an ideal cell line for studying early renal development (Valerius et al., 2002). We found that *Rik* had high basal expression in MK3 cells, so MK3 cell line was selected to construct a *Rik* knockout cell model. To achieve genetic knockout of *Rik*, sgRNAs were designed to target exon 4 and exon 5 of *Rik* based on the start codon, exon location, exon size, and gRNA scores. A YKO-LV005-T2A-EGFP-HygroR vector (Ubigene, China) was used to clone the sgRNA targeting the body of *Rik*, and Cas9 was cloned into a YKO-LV005-T2A-Puro vector (Ubigene, China). MK3 cells were first infected with Cas9 lentivirus and screened with puromycin (Beyotime Biotechnology, China). Subsequently, the MK3-Cas9 cell line was infected with the sgRNA lentivirus and screened with hygromycin B (Solarbio, China). Positive cells were separated 14 days after infection by single-cell deposition into 96-well plates to identify cells that expressed both gRNAs and EGFP. Primers were designed to target exon 4 and exon 5 in the *Rik* region and are listed in Supplementary Table S3. Each single-cell clone was grown and examined using genomic PCR and qRT‒PCR.

### 2.13 Quantification and statistical analysis

Microsoft Excel was employed to analyze the experimental data (*t*-test). The GraphPad Prism 8.0 and Adobe Illustrator software programs were used to produce and design the experimental graphs, and the *p*-value is employed to denote significance (0.05 was the critical value). The information is presented as the mean ± SEM.

## 3 Results

### 3.1 Characterization of lncRNA *Rik* and impaired spermatogenic ability of *Rik* ^*PB/PB*^;*Hoxb7* mice

*Rik* is a lncRNA that has been studied rarely. At present, the NCBI database shows two transcripts of *Rik*, with sizes of 1926 bp and 386 bp. To determine the actual transcript sequence of *Rik* in the mouse model in this study, we first performed a RACE assay.

Our previous study described in detail the spatiotemporal trend of *Rik* expression at various stages of embryonic kidney development in the *Rik* animal model. In *Rik* ^*+/+*^;*Hoxb7* mice, the expression of *Rik* was relatively high from E12.5 to E15.5, peaked at E12.5, progressively declined, and dropped to the lowest level after birth; an overall low expression level of *Rik* was observed. In contrast, *Rik*’s expression level was much higher in *Rik* ^*PB/PB*^;*Hoxb7* mice (Tan et al., 2021).

In addition to observing abnormal phenotypes and expression levels in the neonatal kidneys of *Rik* ^*PB/PB*^;*Hoxb7* mice, we also noticed an interesting phenomenon: mating with homozygous male mice often resulted in a decreased litter size (Supplementary Figure S1A). The observation data revealed that within 3 months, the 3-cage wild-type male mouse group produced a total of 15 litters of mice, whereas the 3-cage homozygous male mouse group produced only 6 litters. Thus, the number of litters per cage per month for homozygous male mice was 0.67, much smaller than that for the wild-type male mice (1.67) (Supplementary Figure S1B). This finding also suggested that the reproductive level was lower in the homozygous male mouse group. The number of sperm was significantly decreased (Supplementary Figure S1C), but the morphology of sperm was generally normal (Supplementary Figure S1D). The testicular and epididymal pathology of adult *Rik* ^*PB/PB*^;*Hoxb7* mice was not significantly different from that of *Rik* ^*+/+*^;*Hoxb7* mice according to HE staining (Supplementary Figure S1E). These results suggest that the *Rik* ^*PB/PB*^;*Hoxb7* mice are fertile but that their spermatogenic ability is affected.

Moreover, the NCBI database indicated that testicular tissue had a greater relative amount of *Rik* expression than kidney tissue (data from http://www.informatics.jax.org/marker/MGI:1921708), so we selected the testis tissues of *Rik* ^*+/+*^;*Hoxb7* mice for RACE. Full-length *Rik* was successfully obtained by rapid amplification of the 5′ end, 3’ end and partial cDNA sequence, with a length of 1,547 bp (Supplementary Figure S2A and Supplementary Figure S2B). Blast comparison with 1926 bp in the NCBI database showed that the lncRNA *Rik* mainly matched the exon 5 fragment (data from https://blast.ncbi.nlm.nih.gov/Blast.cgi). In earlier research, the subcellular distribution of *Rik* in MPTCs was determined through nuclear and cytoplasmic fractionation, and the results showed that *Rik* resided primarily in the nucleus (Tan et al., 2021). FISH analysis of the embryonic kidney tissues of E12.5 *Rik* ^*PB/PB*^;*Hoxb7* mice confirmed this cytoplasmic distribution (Supplementary Figure S2C). The differences in histological and cytological localization suggest that *Rik*, as a lncRNA, may have a relatively broad mode of action, possibly from both the cytoplasm and nucleus. Therefore, we re-employed the sequence analysis program ORF Finder from NCBI, the results of which showed that the predicted proteins were all less than 50 amino acids in length (Supplementary Figure S2D). The coding probability of *Rik* calculated by the Coding-Potential Assessment Tool (CPAT) was as low as 0.048 (Wang et al., 2013) (Supplementary Figure S2E). In summary, we confirmed that the lncRNA *Rik* is a noncoding RNA.

### 3.2 Twenty-eight differentially expressed genes associated with embryonic development are selected through bioinformation analysis

**FIGURE 1:**
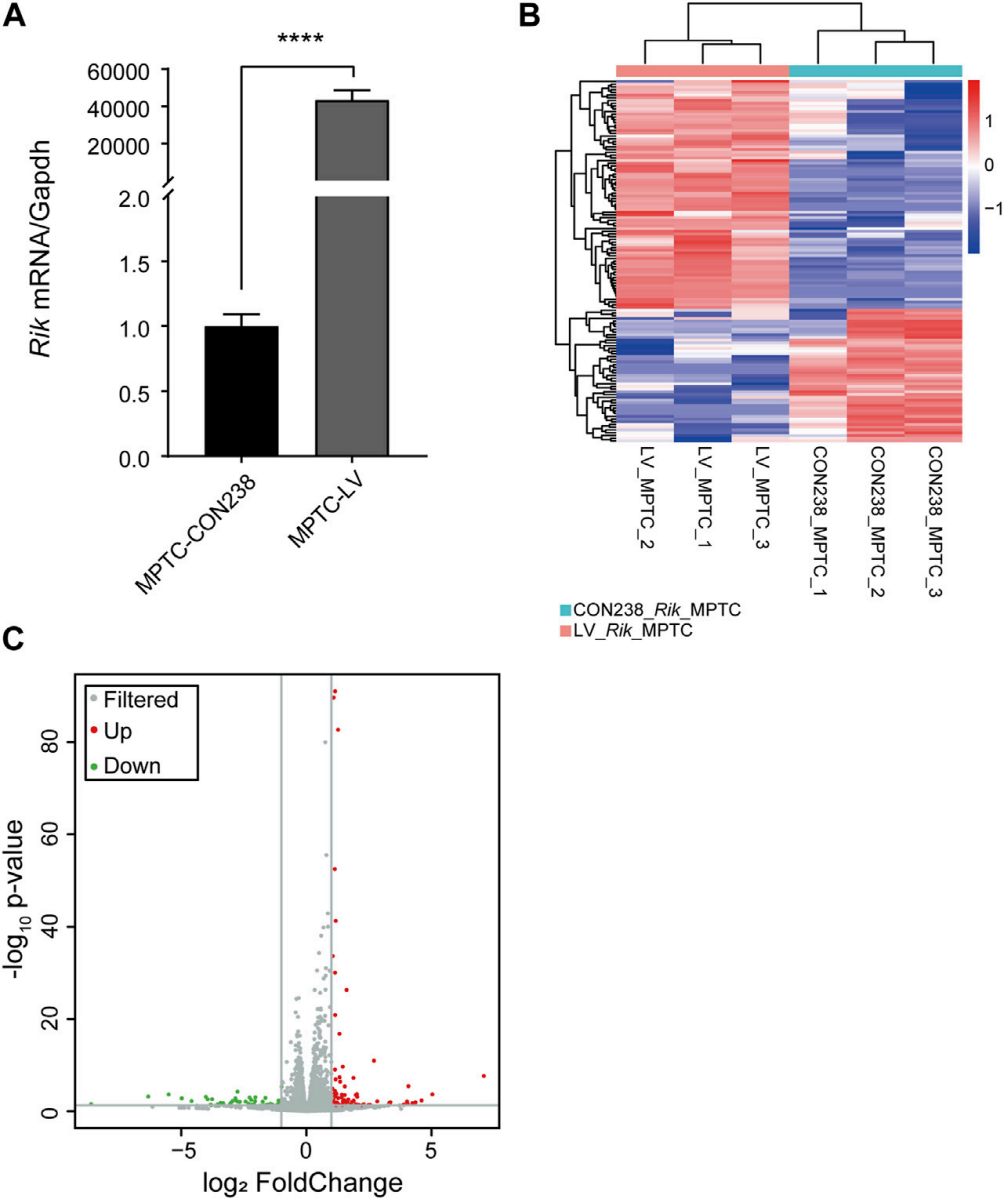
Construction of the MPTCs line with *Rik* overexpression and RNA-seq analysis. **(A)** The degree of upregulation of *Rik* in the MPTCs line was confirmed via qRT‒PCR. **(B)** Heatmap of the differentially expressed genes in the MPTCs line overexpressing *Rik*. Protein-coding genes with a high level of expression are highlighted in red, and blue represents the relatively weakly expressed protein-coding genes. **(C)** Volcano plot of the genes in the MPTCs line whose expression differed in response to overexpression of *Rik*. Genes with no discernible differences are indicated by the color gray, whereas those with discernible differences are indicated by the colors red and green. *p* < 0.05, |log2(fold change) | >1. ****, *p* < 0.0001.

Then, we constructed an MPTCs line overexpressing *Rik* based on the transcript sequence of *Rik* 1,547 bp. *Rik* is a long noncoding RNA, so qRT‒PCR is appropriate to verify the effect of *Rik* overexpression. The results showed that *Rik* was increased by approximately 43,356-fold in MPTCs (Figure 1A). No molecular identification method corresponding to structural malformations of renal development has been reported, so renal development phenotype and function detection at the cellular and molecular levels has not been carried out. To investigate how *Rik* overexpression causes renal hypoplasia, we first performed RNA-seq on the MPTCs line with *Rik* overexpression.

In total, 133 genes were differentially expressed in the MPTCs line overexpressing *Rik* compared with the control line, including 84 genes with increased expression and 49 genes with decreased expression. A differential gene expression map of the overexpression cell model is shown in Figure 1B. The volcano map was drawn based on the -log_10_ (*p*-value) and -log_2_ (fold-change) values of the distinctively expressed mRNAs (Figure 1C).

To determine whether there are developmental signaling pathways related to the above differentially expressed genes, we first conducted pathway analysis of the differentially expressed genes using the KEGG database in the MPTCs line with *Rik* overexpression. We discovered that upregulated differentially expressed genes were strongly concentrated in the immune system, viral infectious disease, signal transduction categories, and organ development and degeneration categories (Supplementary Figure S3A). KEGG enrichment analysis of the downregulated differentially expressed genes revealed that the immune system, organ development and degeneration, and signal transduction categories were all affected (Supplementary Figure S3B).

GO analysis was also performed on upregulated and downregulated differentially expressed genes in the MPTCs line with *Rik* overexpression. As shown in Supplementary Figure S4A, specifically in areas such as actin filaments, elevated genes were primarily involved in immunological defense, cell proliferation, and other functions. Actin is closely related to UB branch morphogenesis (Kuure et al., 2010). The downregulated genes with differential expression were primarily connected to transcription factor binding activity (Supplementary Figure S4B). Next, we comprehensively analyzed the total differences in gene expression in the MPTCs line using GO enrichment analysis. The main enriched functions of the differentially expressed genes were apoptosis, organ morphogenesis, epithelial cell differentiation, signal receptor connection and the cell cycle (Supplementary Figure S5). Given these results and those of the above bioinformatics analysis, we selected a total of 28 differentially expressed genes that may be related to development.

### 3.3 Differentially expressed genes related to the Wnt signaling pathway

**FIGURE 2:**
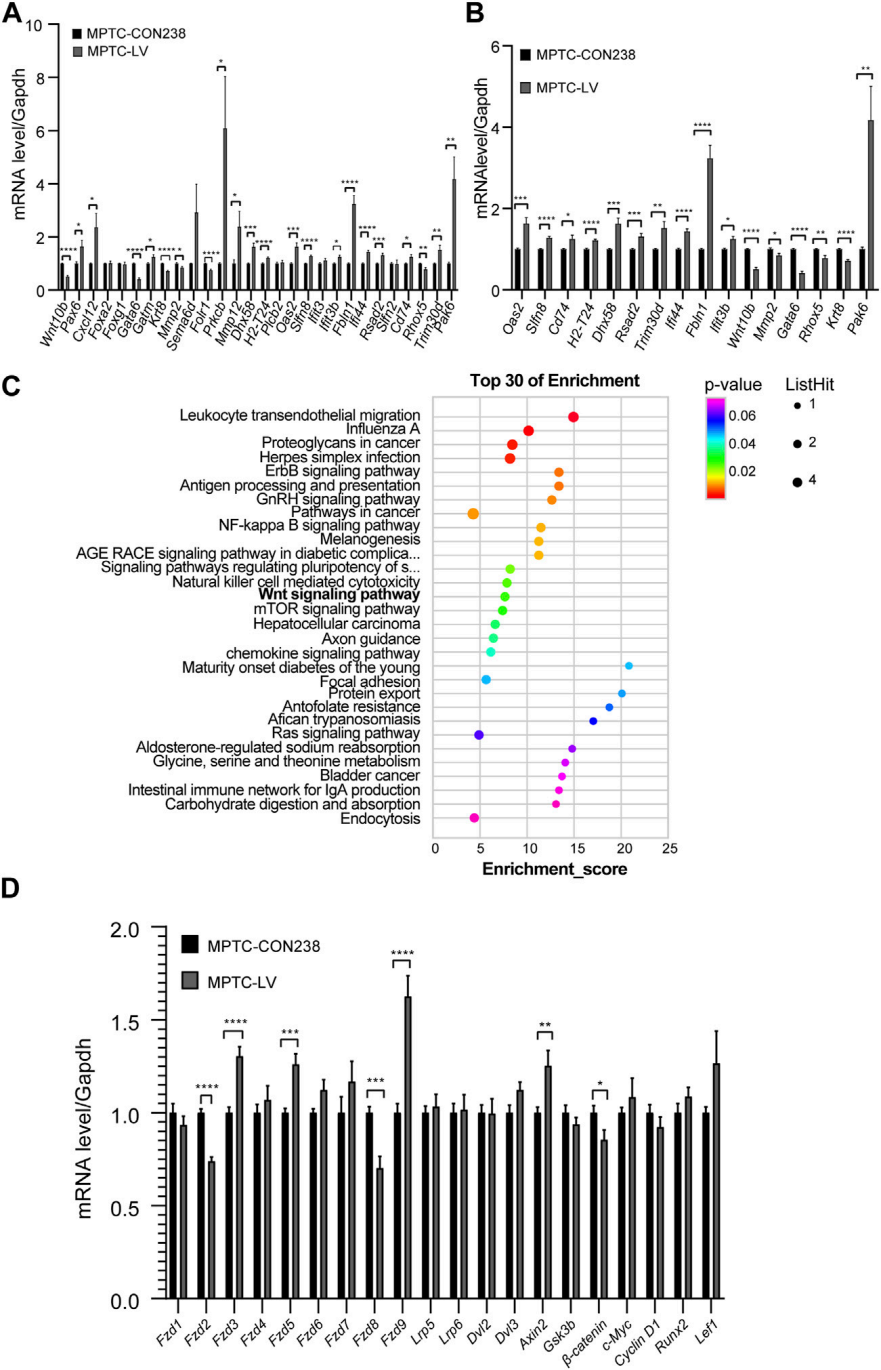
Screening and validation of molecular expression levels in MPTCs overexpressing *Rik*. **(A)** In the MPTCs line overexpressing *Rik*, qRT‒PCR was utilized to confirm the expression patterns of 28 differentially expressed genes. **(B)** Sixteen genes among the 22 differentially expressed genes had the same expression trend as in RNA-seq in the MPTCs line with *Rik* overexpression. **(C)** Results of the study of differentially expressed genes using KEGG. The enrichment score is represented on the figure’s horizontal axis. The bubble color shifts from purple to blue to green to red as it becomes bigger, indicating that there were more differentially expressed protein-coding genes linked to the term. Greater importance is indicated by a lower enrichment *p*-value. **(D)** Molecular expression patterns of Wnt/β-catenin signaling pathway members in the MPTCs line with *Rik* overexpression were confirmed by qRT‒PCR. (n = 3) *, *p* < 0.05, **, *p* < 0.01, ***, *p* < 0.001, ****, *p* < 0.0001.

The expression levels of 28 genes in the MPTCs line with *Rik* overexpression were checked using qRT‒PCR. The findings revealed that 22 genes had significantly varied expression levels, and 16 genes were upregulated, including *Pax6*, *Cxcl12*, *Gatm*, *Prkcb*, *Mmp12*, *Dhx58*, *H2-T24*, *Oas2*, *Slfn8*, *Ifit3b*, *Fbln1*, *Ifi44*, *Rsad2*, *Cd74*, *Trim30d*, and *Pak6*, whereas 6 genes were downregulated, including *Wnt10b*, *Gata6*, *Krt8*, *Mmp2*, *Folr1*, and *Rhox5* (Figure 2A).

Among the 22 differentially expressed genes, 16 had the same expression trend in qRT‒PCR and RNA-seq, 11 of which were upregulated, namely, *Dhx58*, *H2-T24*, *Oas2*, *Slfn8*, *Ifit3b*, *Fbln1*, *Ifi44*, *Rsad2*, *Cd74*, *Pak6*, and *Trim30d*, and 5 of which were downregulated, namely, *Wnt10b*, *Gata6*, *Krt8*, *Mmp2*, and *Rhox5* (Figure 2B).

We repeated the KEGG enrichment analysis for these genes and found that the Wnt signaling pathway was among the highlighted pathways (Figure 2C). It has been claimed that development, particularly renal development, is intimately tied to the Wnt signaling pathway (Wang et al., 2018).

### 3.4 Abnormal expression levels of key molecules in the Wnt/β-catenin signaling pathway in the MPTCs line with *Rik* overexpression

Among the aforementioned 16 genes, *Wnt10b* is a ligand of the Wnt/β-catenin signaling pathway, which has been connected to chronic kidney disease and renal development (Wend et al., 2012). By using qRT‒PCR on the MPTCs line that had *Rik* overexpression, we further validated the transcriptional activity of molecules connected to the Wnt/β-catenin signaling pathway. As shown in Figure 2D, in the frizzle family, *Fzd3* (1.30 times), *Fzd5* (1.26 times) and *Fzd9* (1.62 times) were upregulated, while *Fzd2* (0.73 times) and *Fzd8* (0.70 times) were downregulated, with *Fzd8* exhibiting the greatest decrease in expression. In addition, the expression of *Axin2*, a part of the *β-catenin* inhibitory complex, was increased (1.25 times), but *β-catenin*, a crucial downstream gene of the Wnt/β-catenin pathway, was expressed less (0.93 times).

### 3.5 Decreased expression levels of *Wnt10b*, *Fzd8* and *β-catenin* in the Wnt/β-catenin signaling pathway of *Rik* ^*PB/PB*^ *;Hoxb7* mice

**FIGURE 3:**
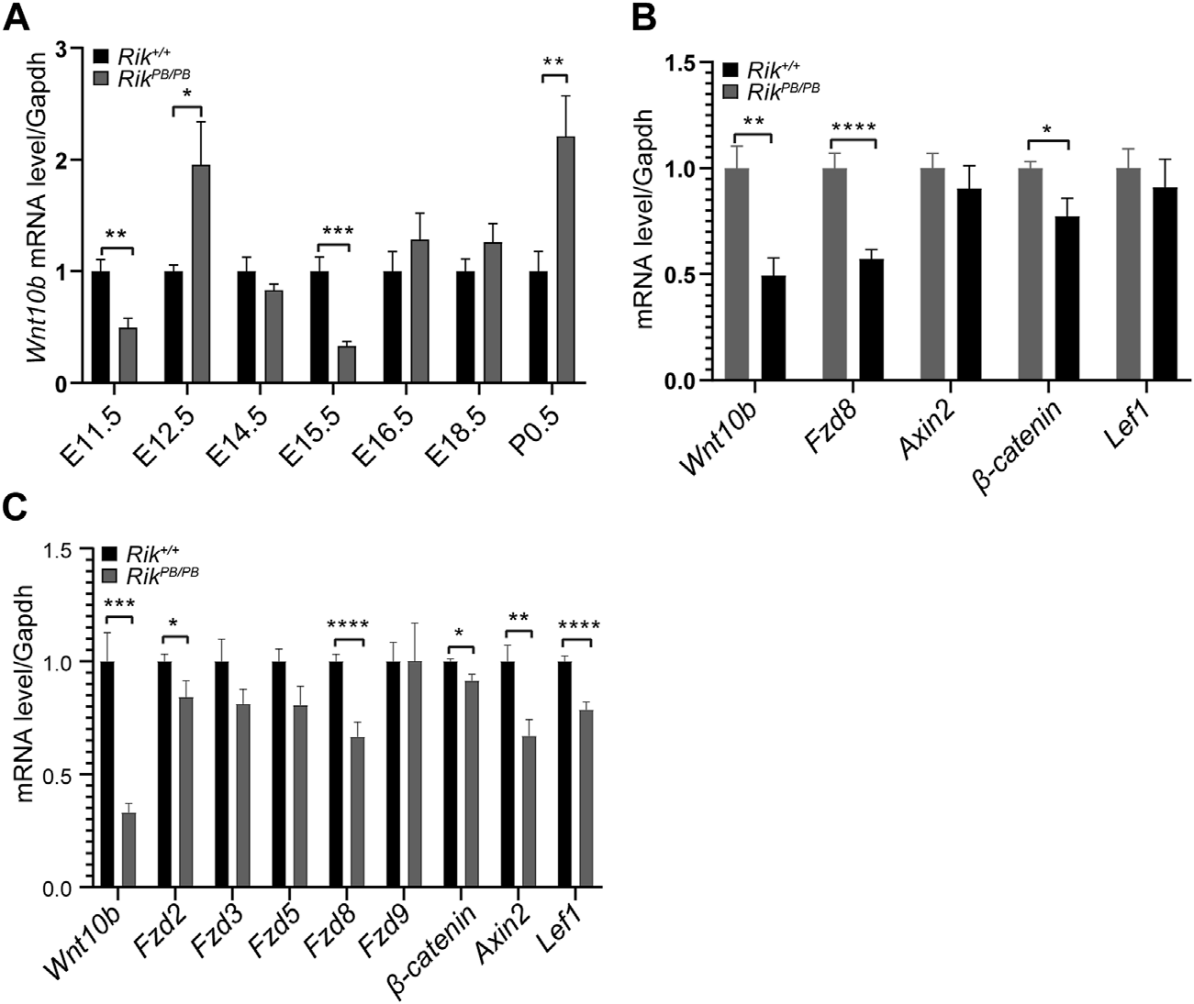
Expression levels of differentially expressed molecules in the embryonic kidneys of *Rik* ^*PB/PB*^;*Hoxb7* mice. **(A)** Expression level of the *Wnt10b* gene at various stages of embryonic kidney development in *Rik* ^*PB/PB*^;*Hoxb7* mice. **(B)** Level of differentially expressed genes in the Wnt/β-catenin pathway in *Rik* ^*PB/PB*^;*Hoxb7* mice at E11.5. **(C)** Level of differentially expressed genes in the Wnt/β-catenin pathway in *Rik* ^*PB/PB*^;*Hoxb7* mice at E15.5. (n = 3) *, *p* < 0.05, **, *p* < 0.01, ***, *p* < 0.001, ****, *p* < 0.0001.

Then, employing qRT‒PCR, we evaluated the activity of the *Wnt10b* gene in the crucial stage of early renal formation in *Rik* ^*PB/PB*^ *;Hoxb7* mice (E11.5, E12.5, E14.5, E15.5, E16.5, E18.5 and P0.5). As shown in Figure 3A, first, we found that the expression of the *Wnt10b* gene in the early T-shaped branching stage of UB was significantly reduced (E11.5); second, in the key branching stage of UB (E12.5-15.5), the *Wnt10b* gene showed a gradual decreasing trend, and the expression at E15.5 was the most obviously reduced, after which the expression of the *Wnt10b* gene gradually increased.

Based on the results shown in Figure 3A, we selected embryonic kidney tissues from E11.5 and E15.5 *Rik* ^*PB/PB*^;*Hoxb7* mice for qRT‒PCR to verify the differentially expressed molecules of the Wnt/β-catenin signaling pathway suggested in the MPTCs line with *Rik* overexpression. We first verified that the *Wnt10b* gene showed a markedly reduced level of expression at both E11.5 (Figure 3B) and E15.5 (Figure 3C). Next, *Fzd8* and *β-catenin* expression levels were also found to be decreased, consistent with the changes we observed in MPTCs overexpressing *Rik in vitro* (Figures 3B,C).

### 3.6 Upregulated expression levels of *Wnt10b*, *Fzd8* and *β-catenin* in the Wnt/β-catenin signaling pathway in the MK3 cell line with *Rik* knockout

**FIGURE 4:**
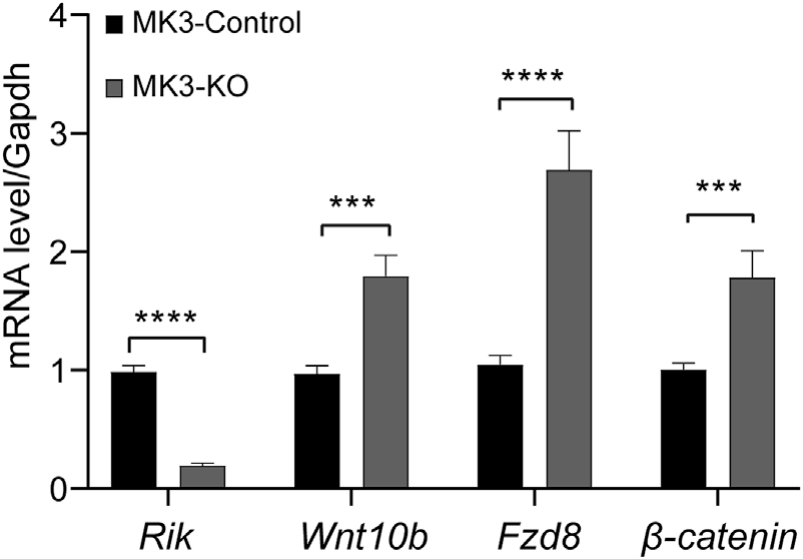
Expression levels of differentially expressed molecules in the MK3 cell line with *Rik* knockout. In the MK3 cell line with *Rik* knockout, qRT‒PCR was utilized to confirm the activity of molecules that were differentially expressed in the Wnt/β-catenin pathway. (n = 6) *, *p* < 0.05, **, *p* < 0.01, ***, *p* < 0.001, ****, *p* < 0.0001.

We constructed a stable *Rik* knockout strain in MK3 cells. We extracted RNA from *Rik* knockout monoclonal cells and found that *Rik*’s expression level was severely reduced, indicating that the stable *Rik*-knockout cell strain was successfully constructed. Furthermore, the expression of critical members of the Wnt/β-catenin signaling pathway (such as *Wnt10b*, *Fzd8* and *β-catenin*) was elevated compared with that in the overexpression cell model and animal model (Figure 4).

## 4 Discussion

**FIGURE 5:**
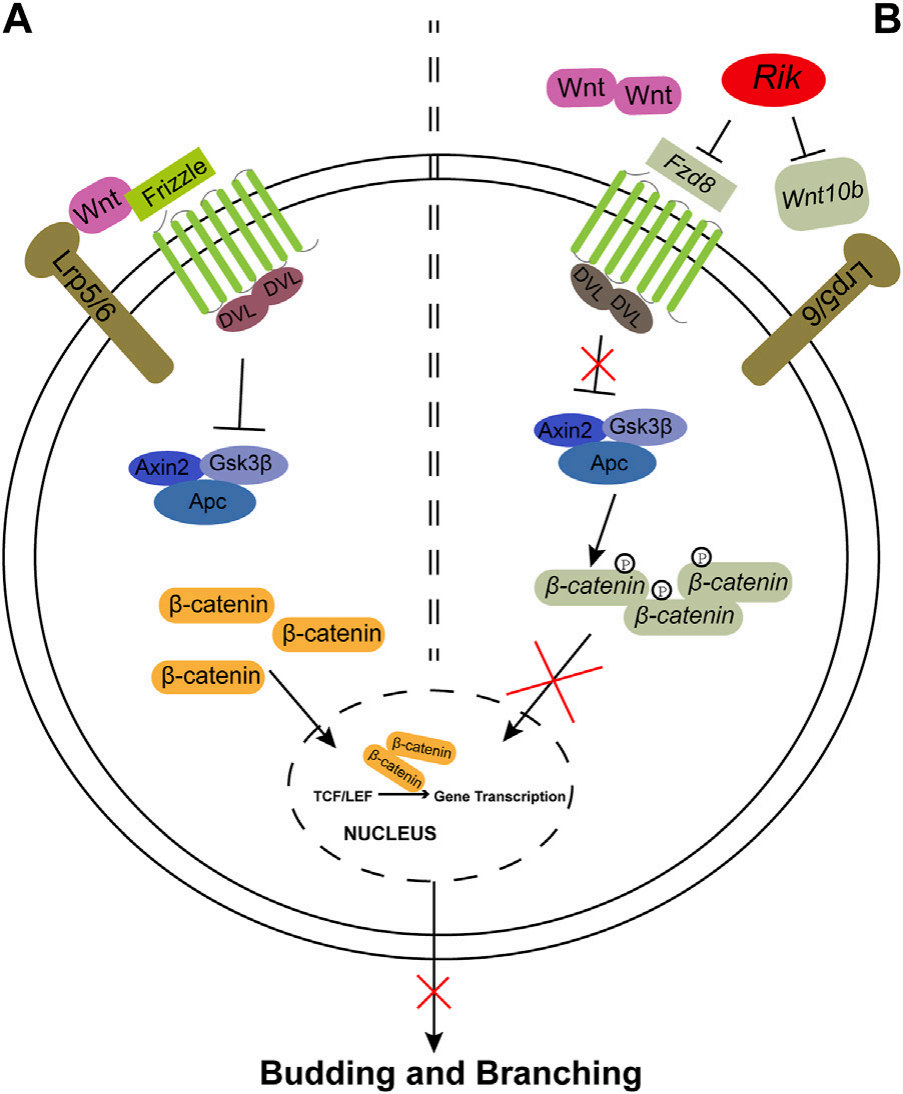
The Wnt/β-catenin signaling pathway controls ureteric bud budding and branching. **(A)**. The Wnt/β-catenin signaling pathway is activated during kidney formation in *Rik* ^*+/+*^ *;Hoxb7* mice. The Wnt ligand binds to the Frizzled (Fzd) family and Lrp5/6 molecules and activates DVL, which is composed of Axin2, Gsk3β and Apc, leading to an increase in β-catenin. Then, β-catenin is transferred to the nucleus to promote the gene transcription process and finally promotes the budding and branching of the UB. **(B)**. *Wnt10b* and *Fzd8* are expressed at lower levels when *Rik* is expressed more strongly, and *Fzd8* may not be able to bind with Wnt ligands, resulting in a decrease in *β-catenin*, which cannot activate the gene transcription process. Eventually, abnormal expression activity of the Wnt/β-catenin pathway may affect the budding and branching process of UB in metanephric development. Observations from the results of this study are marked in gray.

In this study, we successfully constructed a *Rik*-overexpressing MPTCs line and a *Rik*-knockout MK3 cell line by a lentiviral method. Then, we performed GO/KEGG enrichment analysis on RNA-seq data, followed by gene molecular screening and validation, and finally focused on the Wnt/β-catenin signaling pathway. We found that the expression levels of *Wnt10b*, *Fzd8*, and *β-catenin* were reduced when *Rik* was expressed robustly but elevated when *Rik* was knocked out. These results imply that the overabundance of *Rik* might inhibit the Wnt/β-catenin signaling pathway, which might result in renal hypoplasia. A mechanistic hypothesis diagram is shown in Figure 5.

In addition, we measured the *Wnt10b* gene expression level at each stage of kidney development in *Rik* ^*PB/PB*^;*Hoxb7* mice and found that the expression of the *Wnt10b* gene decreased significantly at E11.5 and then showed a clear gradual decrease in the critical stage of UB branching (from E12.5 to E15.5). The expression was lowest at E15.5. The Wnt/β-catenin signaling pathway is reportedly active throughout the early days of kidney development. β-Catenin/TCF signaling activity was first detected near E11.5, gradually concentrated at the end of the UB, and disappeared from the mature UB trunk during the budding and branching stages of the UB from E11.5 to E16.5 (Iglesias et al., 2007). Therefore, E11.5 and E15.5 were the two most abnormal time points. We discovered that *Wnt10b*, *Fzd8* and *β-catenin* all had reduced expression levels at E11.5 and E15.5, with lower levels at E11.5. This difference *in vivo* might suggest that Wnt/β-catenin signaling pathway activity is more inhibited at E11.5 than at E15.5.

Furthermore, although the testicular and epididymal pathology of adult *Rik* ^*PB/PB*^ *;Hoxb7* mice was not significantly different from that of the control groups, we found that the number of sperm, the litter size and pregnancy rate of *Rik* ^*PB/PB*^;*Hoxb7* mice was significantly decreased. Furthermore, the NCBI database indicated that *Rik* was most expressed in mouse testes. Spermatogenesis is a complex and dynamic process which is precisely controlled by genetic and epigenetic factors. Many testis-specific lncRNAs and Wnt/β-catenin signaling pathway were proved to take part in spermatogenesis (Du et al., 2021; Zhou et al., 2022). No literature reports the relationship between *Rik* and spermatogenesis. Our results tentatively suggest that the *Rik* ^*PB/PB*^ *;Hoxb7* mice are fertile but their spermatogenic ability is affected. It might provide some enlightenment for investigating the roles of *Rik* in spermatogenesis. However, more research, such as sperm motility and functions, needs to be investigated and confirmed in the future.

The literature reports that the Wnt/β-catenin signaling pathway not only plays key roles in metabolic processes, tumorigenesis, and stem cell renewal (Russell and Monga, 2018; Raslan and Yoon, 2020; Zhang and Wang, 2020), but also exerts significant regulatory control over growth and branching morphogenesis of UB (E11.5-E16.5), the differentiation of the MM into renal tubules and the establishment of the proximal–distal nephron axis (DasGupta and Fuchs, 1999; Park et al., 2007; Gallegos et al., 2012). The typical Wnt/β-catenin signaling pathway controls the binding of Wnt ligands to Lrp/Fzd family molecules and then cascades to regulate the expression of the key downstream molecule β-catenin, ultimately affecting the transcription of downstream target genes (Clevers and Nusse, 2012; Wang et al., 2018; Schunk et al., 2021). Moreover, studies have found that in Wnt ligand-deficient mice, knockout mice of key molecules of the Wnt/β-catenin signaling pathway, or mice treated with the Wnt/β-catenin signaling pathway inhibitor DKK1, most mice exhibit renal hypoplasia and fewer UB branch tips (Majumdar et al., 2003; Satow et al., 2004; Pietilä et al., 2016). The phenotype of deficient mice is comparable to the reduction in UB branch tips discovered in our prior investigation (Tan et al., 2021).

Our previous study found that the expression of *Bmp4* and its downstream phosphorylated Smad1/5/8 was significantly reduced in *Rik* ^*PB/PB*^ mice (Tan et al., 2021). BMP4 belongs to the transforming growth factor beta (TGF-β) superfamily, and studies of the mechanisms of renal hypoplasia underscore a crucial role of the BMP/SMAD signaling for renal morphogenesis in animal models (van der Ven et al., 2018). AhR is a ligand-activated transcription factor and binding to the AhR has been demonstrated to trigger the phosphorylation of the TGF-β1 superfamily. In addition, studies have suggested that there may be functional crosstalk between AhR and the TGF-β/BMP/SMAD pathway (Liu et al., 2020). Our present work underlines the significance of the Wnt/β-catenin signaling pathway. According to reports, the antagonist gremlin 1 reduces BMP4 activity in the mesenchyme, allowing outgrowth of UBs and creation of autoregulatory GDNF/WNT11 feedback signaling, which is necessary for the start of metanephric kidney development (Michos et al., 2007). Moreover, it has been reported that there may be crosstalk between BMP/SMAD and Wnt/β-catenin signaling pathways during the differentiation of the stem cells (Wang et al., 2022). Therefore, we propose the idea that renal hypoplasia may not only be caused by the inactivation of Wnt/β-catenin signaling pathway in *Rik* ^*PB/PB*^;*Hoxb7* mice, but also by AhR and TGF-β/BMP/SMAD signaling pathway. More studies are needed to be investigated.

This research had several limitations. First, there remains no evidence to directly indicate that the decline in binding of *Fzd8* to its receptor is due to the regulation of *Rik*, nor can we prove that *Wnt10b* directly regulates *Fzd8.* Second, our current research focused mainly on the regulation of Wnt/β-catenin signaling pathway. However, as shown in Supplementary Figures S3–S5, many treasures remain to be investigated, such as actin filaments, the immune system and other signal transduction pathways, which need to be further investigated. Thirdly, due to the small size of the embryonic kidney tissues, Western blotting and *in situ* hybridization experiments could not be performed to validate the expression of the Wnt/β-catenin pathway members in this study.

These results imply that excessive *Rik* expression may inhibit the Wnt/β-catenin pathway, resulting in renal hypoplasia. Generally, such research may help shed light on CAKUT causes and processes and offer guidance for creating new prophylactic and therapeutic strategies.

## Data Availability

The datasets presented in this study can be found in online repositories. The names of the repository/repositories and accession number(s) can be found in the article/Supplementary Material.
