# Supplementary material for: Overexpression of long noncoding RNA 4933425B07Rik leads to renal hypoplasia by inactivating Wnt/β-catenin signaling pathway
Source: Front Cell Dev Biol. 2023 Oct 17;11:1267440. doi: 10.3389/fcell.2023.1267440 (PMC10616775; doi:10.3389/fcell.2023.1267440)
Supplement: Supplementary file 1 [file Table6.DOCX]

Supplementary Material

1. **Supplemental Figures and Tables**
2. **Figures**

**
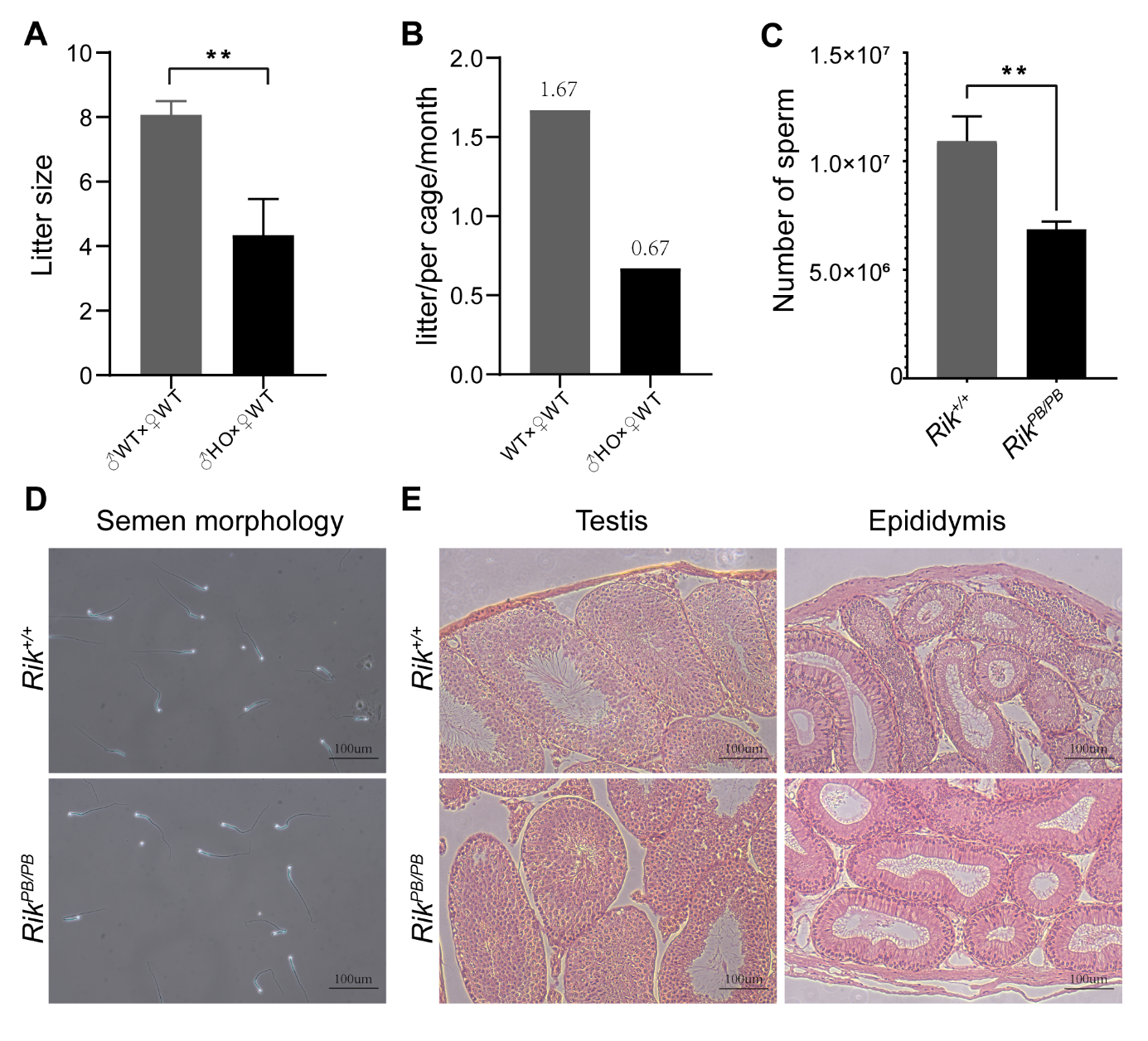
**

**Supplemental Fig. S1 Initial assessment of reproductive capacity in *Rik^+/+^*;*Hoxb7 male mice and Rik^PB/PB^*;*Hoxb7* male mice. (A)** The litter size of the mating group of *Rik^PB/PB^*;*Hoxb7* male mice (♂HO) and *Rik^+/+^*;*Hoxb7* female mice (♀WT) (n=3) was significantly lower than that of *Rik^+/+^*;*Hoxb7* male mice (♂WT) and *Rik^+/+^*;*Hoxb7* female mice (♀WT) (n=3). (**B**) The number of litter/per cage/month of the mating group of *Rik^PB/PB^*;*Hoxb7* male mice (♂HO) and *Rik^+/+^*;*Hoxb7* female mice (♀WT) (n=3) was lower than those of *Rik^+/+^*;*Hoxb7* male mice (♂WT) and *Rik^+/+^*;*Hoxb7* female mice (♀WT) (n=3). (**C**) The sperm numbers of *Rik^PB/PB^*;*Hoxb7* male mice (n=5) were significantly lower than those of *Rik^+/+^*;*Hoxb7* male mice (n=4) at the same age (3 months). (D) Sperm morphology analysis (semen smear) of *Rik^PB/PB^*;*Hoxb7* male mice and *Rik^+/+^*;*Hoxb7* male mice. (E) Hematoxylin-eosin staining of testicles and epididymis of *Rik^PB/PB^*;*Hoxb7* male mice and *Rik^+/+^*;*Hoxb7* male mice. **, P < 0.01. Scar bars, 100 µm in (D-E).

**
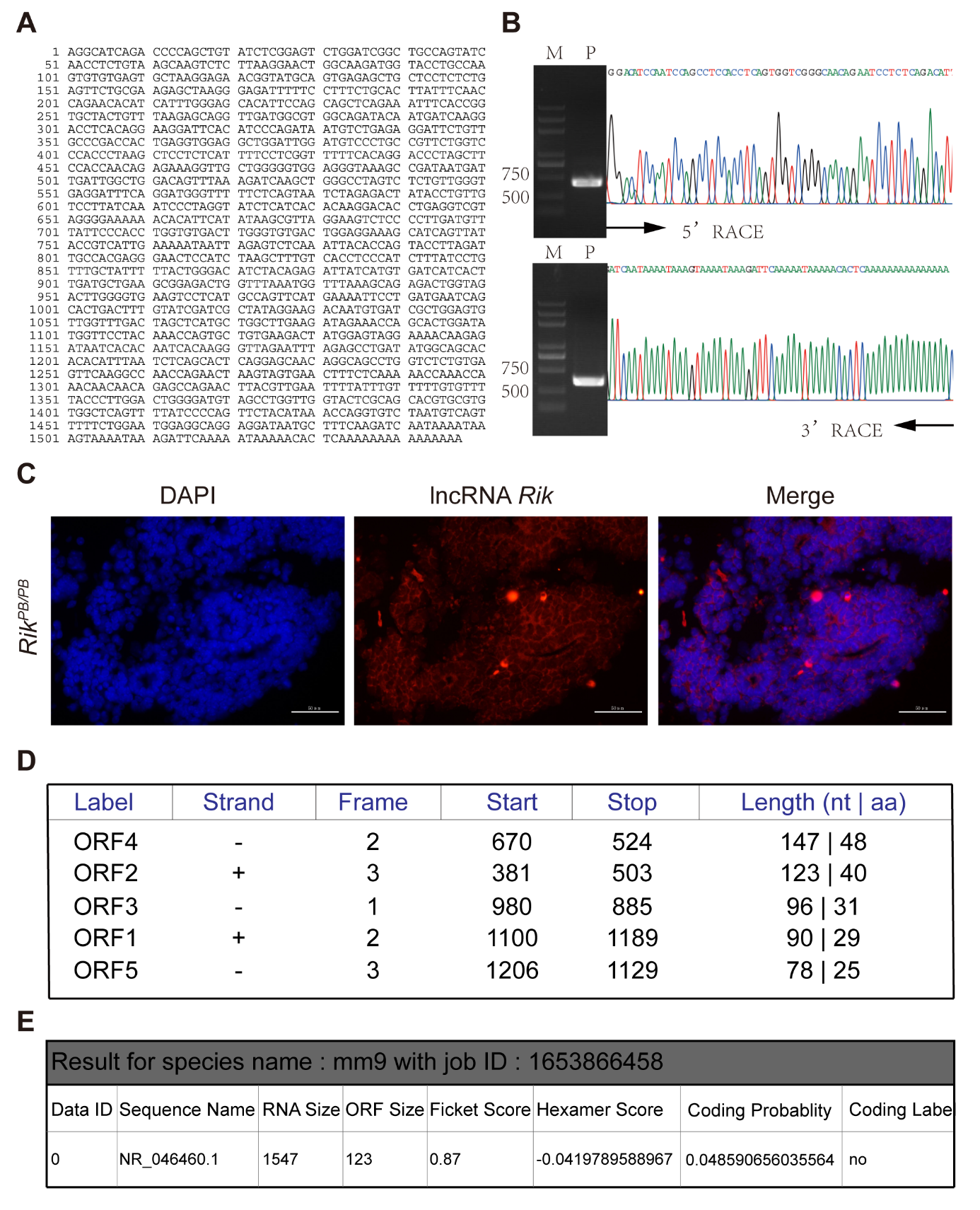
**

**Supplemental Fig. S2** **Identification of the lncRNA *Rik* and analysis of its noncoding nature.** **(A)** Nucleotide sequence of full-length lncRNA *Rik* determined by rapid amplification of cDNA ends (RACE). **(B)** 5’ and 3’ RACE assays on testis samples to detect the whole sequence of the lncRNA *Rik*. Left; cropped gel electrophoresis images of PCR products from the 5’-RACE and 3’-RACE assays. M: DL5000 marker. From bottom to top: 100, 250, 500, 750, 1000, 1500, 2000, 3000, and 5000 bp. P: PCR products. The product in the 5’-RACE lane is 403 bp, and the product in the 3’-RACE lane is 298 bp. Right: sequencing of PCR products. The top wells were cropped, and the original unprocessed gel electrophoresis images of Supplemental Fig. S1C are included at the end of the Supplemental Materials. **(C)** Fluorescence in situ hybridization (FISH) assay (red) to examine the expression and localization of the lncRNA *Rik* in *Rik^PB/PB^*;*Hoxb7* kidney tissue at E12.5. Scale bars, 50 μm. **(D)** Putative proteins encoded by the lncRNA *Rik* as predicted using ORF Finder. **(E)** Coding potential of the lncRNA *Rik* predicted by CPAT.


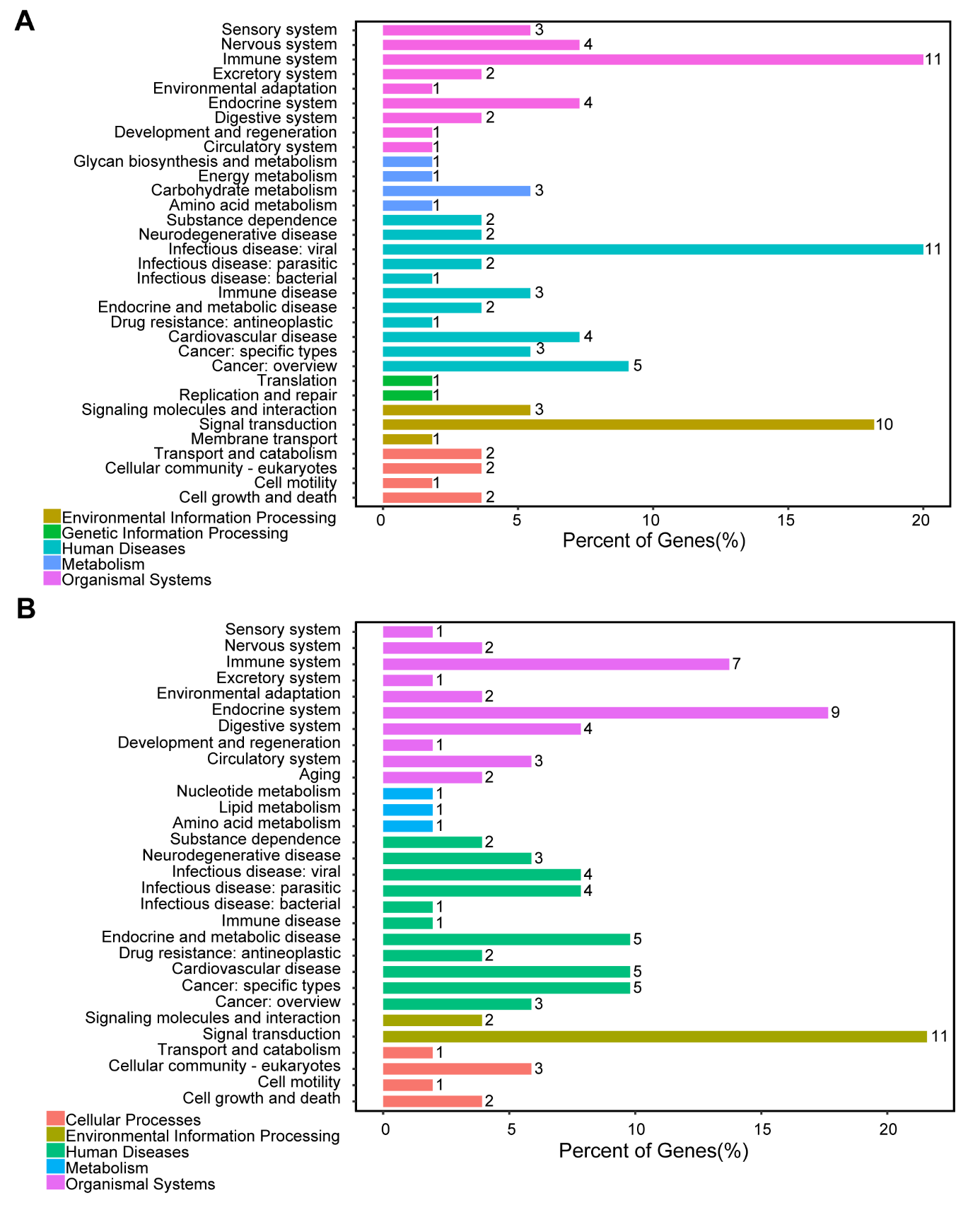


**Supplemental Fig. S3** **KEGG enrichment analysis of differentially expressed genes** **in the MPTCs line with *Rik* overexpression.** **(A)** KEGG enrichment analysis of upregulated differentially expressed genes. **(B)** KEGG enrichment analysis of downregulated differentially expressed genes. The horizontal axis is the ratio (%) of the total number of genes annotated to each level-2 pathway (differentially expressed genes) and all genes annotated to KEGG pathways (differentially expressed genes). The vertical axis shows the name of the level-2 pathway, and the number on the right side of the column is the number of differentially expressed genes annotated to this level-2 pathway.


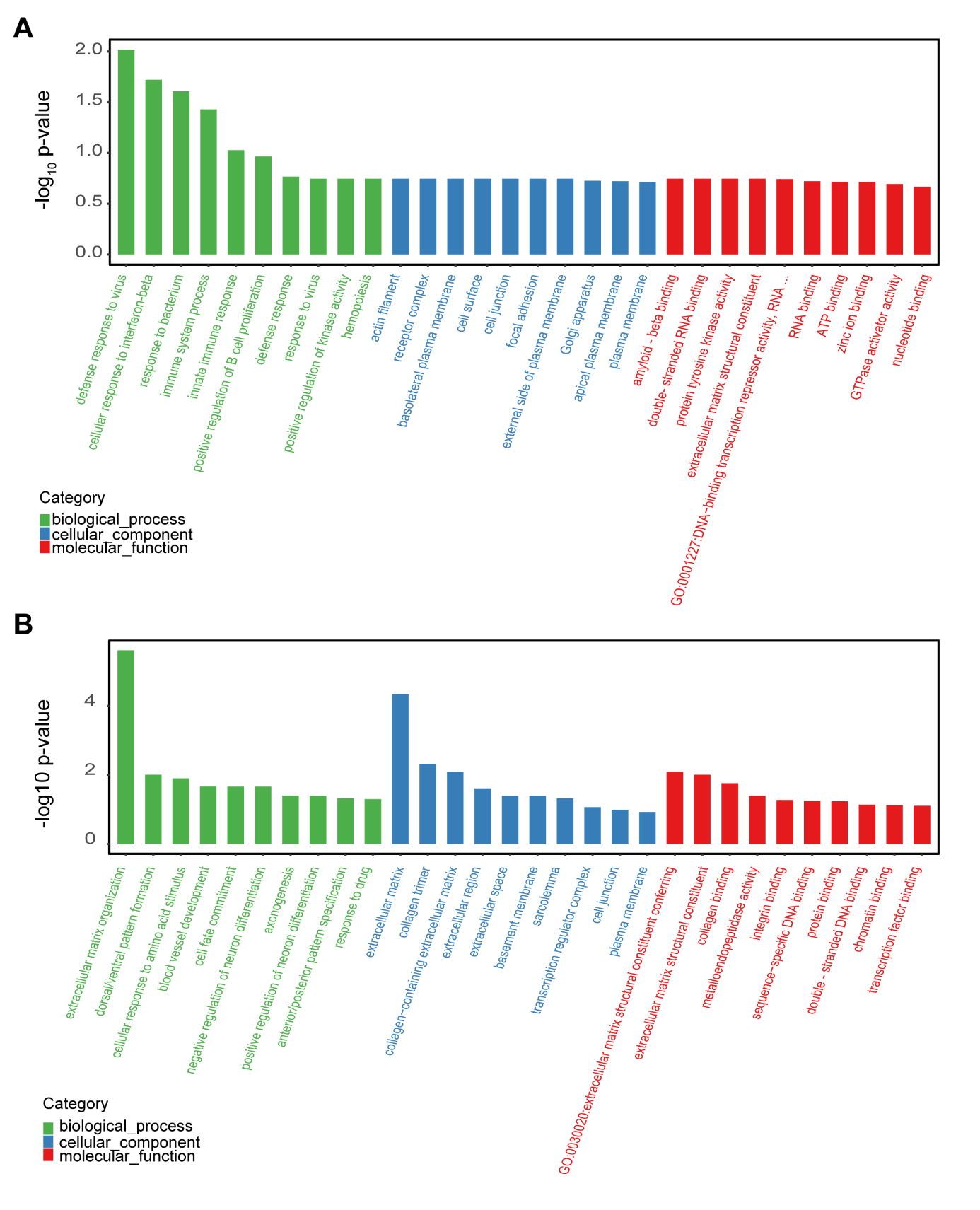


**Supplemental Fig. S4** **GO enrichment analysis of differentially expressed genes in the MPTCs line with *Rik* overexpression.** **(A)** Top 30 GO terms of upregulated differentially expressed genes. **(B)** Top 30 GO terms of downregulated differentially expressed genes. The GO terms with more than 2 corresponding differentially expressed genes in the three classifications were screened, and the top 10 terms were sorted from large to small according to the -log_10_ p value corresponding to each term. The horizontal axis is the name of the GO term, and the vertical axis is the -log_10_ p value.


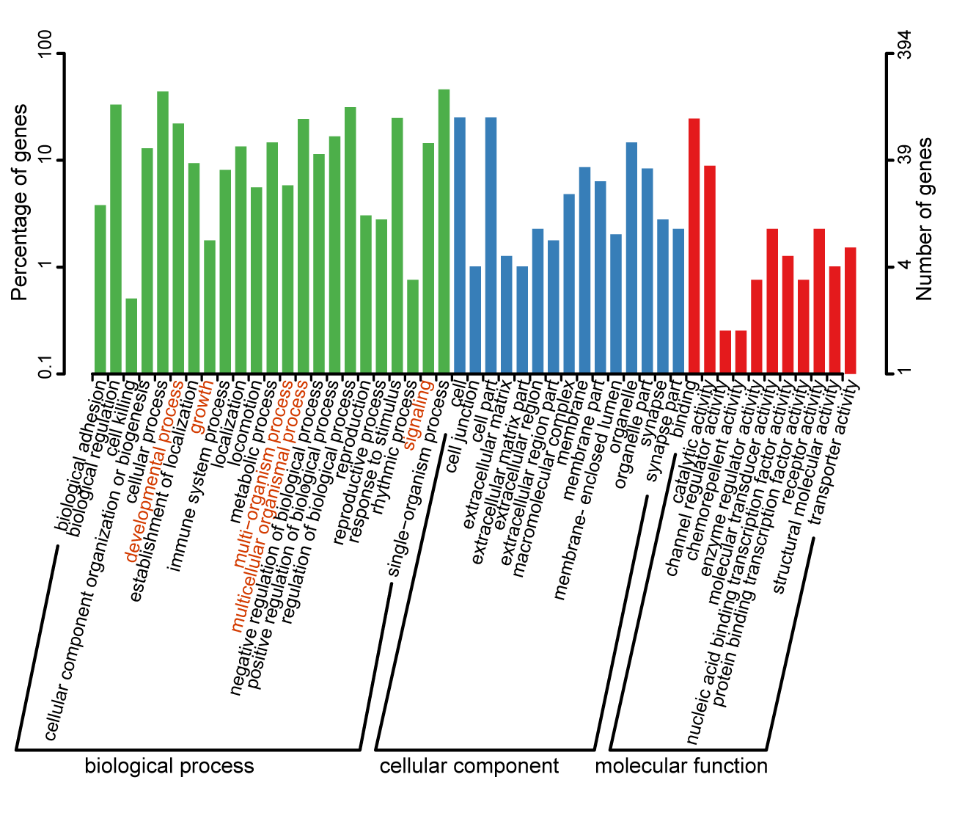


**Supplemental Fig. S5 Comparison of the distribution of differentially expressed genes at GO level 2 in the MPTCs line with *Rik* overexpression.** **(A)** GO classification of the total differentially expressed genes in the MPTCs line with *Rik* overexpression. GO level 2 includes biological process, cellular component and molecular function terms. The column shows the GO level-2 terms enriched for differentially expressed genes, the horizontal axis shows the name of the term, and the vertical axis shows the number and percentage of genes of the corresponding term.

1. **Tables**

**Supplemental Table. S1 Genotyping primers.**

| **Gene** | **Forward Primer/Reverse Primer** | **WT band size (bp)** | **PB band size (bp)** |
| --- | --- | --- | --- |
| *Rik* | L1A：  ACTAATGGTAGTAGTCCTGCTAGGC | 535 | 817 |
| *Rik* | R1A：  ACTCACAGACAATTTACCTTCAGGC |  |  |
| *Rik* | LB2：  CTGAGATGTCCTAAATGCACAGCG |  |  |
| *Hoxb7* | F：AGAGCAAGCCAAAGGTGAG | 500 |  |
| *Hoxb7* | R：CTTGATGCCGTTCTTCTGC |  |  |

Oligomers listed in 5’- to 3’- orientation.

**Supplemental Table. S2 qRT-PCR primers.**

| **Gene** | **Forward Primer** | **Reverse Primer** |
| --- | --- | --- |
| *Rik* | TTGGGTGTGACTGGAGGAAA | CAAACAGGATAAAGATGGGAGGT |
| *Gapdh* | TGTTCCTACCCCCAATGTGTCC | GGAGTTGCTGTTGAAGTCGCAG |
| *Wnt10b* | ACTGGTGCTGTTATGTGCTGTGTG | CTTCTCCTCCTCGTCGTGCCTAG |
| *Pax6* | AGGGCAATCGGAGGGAGTAAGC | TCTGTCTCGGATTTCCCAAGCAAAG |
| *Cxcl12* | GGATGGGTTGAAGTAGGATGTGCTC | AGGTCTTGGTGCTCAGGAGGTG |
| *Foxa2* | CTGAAGCCCGAGCACCATTACG | GGTGGTGGCTGTGGTGATGTTG |
| *Foxg1* | ATCAGGCAGAGTCCCGAGAAGC | CAGGTTGTGGCGGATGGAGTTC |
| *Gata6* | CCGTGCGACAGGATTCTTGGTG | GCCATCTGGACTGCTGGACAATATC |
| *Gatm* | AACAGCACCCAAGCCCACAATG | CGAATGAAGTCAGCAGCATCAAAGC |
| *Krt8* | GAGACCAAGTGGAGCCTGTTGC | CGGCGGAGGTTGTTGATGTAGC |
| *Mmp2* | ACCATGCGGAAGCCAAGATGTG | AGGGTCCAGGTCAGGTGTGTAAC |
| *Sema6d* | ACGGTGTACGGTGGGAAGTC | GGACATTCATGTGGACCATCTG |
| *Folr1* | ACACAAGCCAGGAAGCACATAAGG | ATTCCGATGTCATAGTTCCGCAGTG |
| *Prkcb* | ACTCGAACGCAAGGAGATTC | CAGGAGGTGTTAGGACTGGTG |
| *Mmp12* | GAGTCCAGCCACCAACATTAC | GCGAAGTGGGTCAAAGAC |
| *Dhx58* | TCTGGAGGTGCTGATGGAGAAGG | GTGCTGCCCGCTTAACTAGAGATG |
| *H2-T24* | GAACTCAGGACCTTCGGAAGAACG | GCCAGTGCTTAGTTGTGCCTCTC |
| *Plcb2* | GTTCAACGGGCAGAGTGGCTAC | CGTAATGGAAAGGGTGGTGGCTAC |
| *Oas2* | GAGTTCTCAGACTGCTTCACCACAC | CACTTCTCCTGGCACTGTTCATACC |
| *Slfn8* | CGTGCCCAGACTTGACCATC | AACAGAGCACAAGCAGCCTC |
| *Ifit3* | GCAGTTCTCCGTGGATGCTC | CTCAGCAGTTTCAGGGCCAG |
| *Ifit3b* | AGAAGCCCAAGGACCCAGAG | CCTGCTTCAGAGCATCCACG |
| *Fbln1* | TGACGAGGAGGACCAAGAAGACC | AGCCCACAAAGCAAGAGCAGATC |
| *Ifi44* | GAGTTCTGCTGCTGGGTCCTATTG | CTGGTGTGTGATGCTGCCCTTG |
| *Rsad2* | CCTCTGTGAGCATAGTGAGCAATGG | TGTCGCAGGAGATAGCAAGAATGTC |
| *Slfn2* | GCCCTATCGTTCATCCTACCCTTTG | CCCATTCCCGCCAAATCATCCTG |
| *Cd74* | TCCCAGAACCTGCAACTGGA | ATTGGACGCATCAGCAAGGG |
| *Rhox5* | TCCTGCCTTCCGTGGACAAGAG | ATGCTGTTCTTCCGAGTCTTCCTTG |
| *Trim30d* | CTTCAGAGGAGCAGCACAGTTGG | AGAGACCCAGGATTTGTGGAGAGG |
| *Pak6* | CTGTACGCTACTGAGGTGGA | GTACCAGCATCCGATCCAGG |
| *Fzd1* | CACCTGGATAGGCATCTGGT | CAGAAAGCCAGCGATGTAGG |
| *Fzd2* | CCGACGGCTCTATGTTCTTC | TAGCAGCCGGACAGAAAGAT |
| *Fzd3* | AGCGTGCCTATAGCGAGTGT | TCTCTGGGACACCAAAAACC |
| *Fzd4* | CTGCAGCATGCCTAATGAGA | CGTCTGCCTAGATGCAATCA |
| *Fzd5* | TCTTGTCTGCGTGCTACCTG | GGCCATGCCAAAGAAATAGA |
| *Fzd6* | TGTTGGGCTGTCTCTCCTCT | TCTCCCAGGTGATCCTGTTC |
| *Fzd7* | CCATCCTCTTCATGGTGCTT | TGGCCAAAATGGTGATTGTC |
| *Fzd8* | CTGTTCCGAATCCGTTCAGT | CGGTTGTGCTGCTCATAGAA |
| *Fzd9* | AGAGCAACCATGTACTGCTG | GCTCTGCCAAGTCTGAAAAG |
| *Lrp5* | CAGGTGCTTGTGTGGAGAGA | CATGTTGGTGTCCAGGTCAG |
| *Lrp6* | GGTGTCAAAGAAGCCTCTGC | ACCTCAATGCGATTTGTTCC |
| *Dvl2* | AGGAGACTCGGATGAGGATGACAC | CGGTGACACTGCTGAAGGATGAG |
| *Dvl3* | ATGGAACGCACAGGAGGCATTG | GGTCTCCGTGTCATTGTCCAGATTC |
| *Axin2* | GGGTTCTGAAATTCATAGACTAAGA | CGACTGTTCAATAAATATCAGTAA |
| *Gsk3b* | GTTCGGCTACCTTCGGCATTCC | CGAGCGACCTGGATAACCAATGAG |
| *β-catenin* | GCTGCTGTCCTATTCCGAATGTCTG | GGCACCAATGTCCAGTCCAAGATC |
| *c-myc* | GCTCGCCCAAATCCTGTA | AGGACTCGGAGGACAGCA |
| *Cyclin D1* | CGTATCTTACTTCAAGTGCGTG | ATGGTCTCCTTCATCTTAGAGG |
| *Runx2* | TTTAGGGCGCATTCCTCATC | TGTCCTTGTGGATTAAAAGGACTTG |
| *Lef1* | AAATGGGTCCCTTTCTCCAC | TCGTCGCTGTAGGTGATGAG |

Primers utilized to amplify gene. Oligomers listed in 5’- to 3’- orientation.

**Supplemental Table. S3 Knockout cell genotyping primers.**

| **Gene** | **Forward Primer/Reverse Primer** | **WT band size (bp)** | **KO band size (bp)** |
| --- | --- | --- | --- |
| *Rik*-F1 | GAACTTGTTTGGCATAAACCGAG | 3292 | 2800 |
| *Rik*-R1 | GTTAATGTGAGTAACGGAGTCGG |  |  |
| *Rik*-F3 | CAGTACCTTAGATTGCCACGAGG | 1268 | 0 |

Oligomers listed in 5’- to 3’- orientation.
